# Certainty Level of Water Delivery of the Required Quality by Water Supply Networks

**DOI:** 10.3390/ijerph16101860

**Published:** 2019-05-27

**Authors:** Marian Kwietniewski, Katarzyna Miszta-Kruk, Kaja Niewitecka, Mirosław Sudoł, Krzysztof Gaska

**Affiliations:** 1 Department of Water Supply and Wastewater Management, Faculty of Building Services, Hydro and Environmental Engineering, Warsaw University of Technology, 00-661 Warsaw, Poland; marian.kwietniewski@gmail.com (M.K.); katarzyna.miszta-kruk@pw.edu.pl (K.M.-K.); kaja.niewitecka@pw.edu.pl (K.N.); miroslaw.sudol@pw.edu.pl (M.S.); 2 Institute of Water and Wastewater Engineering, Faculty of Energy and Environmental Engineering, Silesian University of Technology, 44-100 Gliwice, Poland

**Keywords:** water distribution system, security of water supply, reliability, water quality in the network

## Abstract

The security of water delivery of the required quality by water supply networks is identified with the concept of reliability. Therefore, a method of reliability evaluation of water distribution of the required quality was developed. The method is based on the probabilistic character of secondary water contamination in the water supply network. Data for the method are taken from monitoring of the water distribution system. The method takes into consideration the number and locations of individual measurement points and the results of the tests of water quality indicators at these points. The sets of measurement points and water quality indicators constitute a matrix research (observation) field in the model. The proposed method was implemented to assess the reliability of a water distribution process with respect to water with the required microbiological quality indicators in a real distribution system.

## 1. Introduction

The basic objective pursued by water supply companies is to provide consumers with water of the required quantity, pressure, and quality safe for its consumption. Water shortages or insufficient pressure are not as dangerous to the security of the water supply as water contamination in the distribution network.

As demonstrated by the results of research studies and observations [1,2,3,4,5,6,7,8,9,10,11,12], despite great efforts employed and concerns held by water supply companies, the quality standards for water supplied to consumers [13] are not always maintained. As a result of randomly occurring incidents, but also not infrequently due to human failures, the water supplied to consumers gets contaminated. There is virtually no possibility of completely securing the water supplied to consumers against potential change of its composition.

Such incidents are currently quite common in water distribution systems worldwide. The biggest threat to the safety of water supplied to consumers is its secondary pollution in water supply networks, which is the last link of the water supply system supplying water to indoor installations in buildings. Other less frequently occurring events, but not less dangerous for the consumers, involve extreme incidents reported at various places in the water supply network, e.g., at water intakes.

As demonstrated in the Guidelines of the World Health Organization, the vast majority of real health problems associated with water are brought about by microbiological contamination. The research studies of Świderska-Bróż [10] demonstrated that even a tiny content of nitrogen compounds in sediments found in water pipes is sufficient to ensure the survival of microorganisms that pose a threat to the sanitary quality of water supplied to customers [14,15].

We can observe from the data available in the ENHIS system (European Environment and Health Information System) that in the years 2000–2007, 354 outbreaks of diseases related to non-performance of drinking water quality parameters were identified and confirmed in 14 European countries, including over 47,617 diagnosed cases of disease. The most common causes of diseases reported in those countries were bacteriological contaminations of water (*Campylobacter*, *Aeromonas* sp., and *Shigella sonnei*)—responsible for 44.9% (163 cases) of outbreaks and 33.3% of diseases. Viruses were confirmed to have been the cause of disease in 136 outbreaks (37.5%) and 49.4% of disease cases, and protozoa caused 17 outbreaks (4.7%) and 9.9% of disease cases [16].

In the years 1981–2010, 57 epidemics were identified in the United States, which caused 9000 disease cases [17,18,19,20,21,22,23,24,25,26,27,28,29]. The most common causes of disease were intestinal pathogens, including bacteria (*Salmonella*, *Campylobacter*, *Shigella*, *Escherichia coli*), protozoa (*Cryptosporidium*, *Giardia*), and viruses [30].

The outbreaks of epidemics that have taken place in highly developed countries are often associated with human errors made during the intake, treatment, and distribution of water. For example, such an incident of water contamination in a water supply network was reported in Finland in the years 2007–2008. The system detected high levels of the indicators of fecal contamination and *Salmonella*, *Campylobacter*, viruses, and *Giardia*. In the population of approximately 30,000 inhabitants, 5000 cases of disease were reported (inflammation of the digestive tract). The outbreak developed due to the error of an employee responsible for the operation of the water supply network. It is estimated that the costs related to the outbreak of the epidemic (treatment, absence from work, etc.) and the undertaken corrective actions amounted to approximately EUR 3.7 million [31,32].

Another example of an epidemic involves the disruption of stable water flow in a distribution network (supplying 18,000 people) which happened in connection with water intake for the purposes of fire fighting in Hemikes and Schelle in Belgium (2010). Based on the tests carried out, symptoms of gastric disorders in a group of 3400 people were reported. *Campylobacter* sp., GI and GII Noroviruses, and Adenono viruses were detected in the feces samples, and fecal contamination indicators were found in water samples [33].

Even the few presented examples demonstrate the level of threat to the safety of the water supply to consumers. Therefore, it is essential to define and articulate the level of this threat as a measure of water supply certainty of the required quality. For this purpose, we can use reliability theory and apply an appropriate model of water supply of the required quality. The assessment of reliability is based on operational research conducted in the natural operating conditions of the water distribution system. The mentioned research is based on the monitoring results of the network water quality, which are then analyzed statistically and stochastically. The most significant factor of monitoring is that it allows us to control and assess the impact of contamination on the supply certainty of water of the required quality.

Regardless of numerous works on the reliability of water distribution systems [34,35,36,37,38,39,40,41], the problem involving the assessment of certainty of the water supply having the required quality has not yet been fully resolved. This issue was discussed in the context of water quality monitoring in water supply networks, e.g., in the work [42], and to some extent this problem was addressed in the publications [32,43,44,45,46,47].

In the work [42], the author developed a monitoring system of the quality of water distributed through the water supply network in order to assess the reliability of water supply of the required quality. The author applied several typical indicators related to the value of water quality parameters. Reliability has been described by several typical indicators adapted to water quality, such as the level of compliance or non-compliance of the water quality indicator with the required standard, the duration of compliance or non-compliance, or the volume of water compliant with the standards. However, the presented methodology is extremely complex and requires an extensive set of data; this significantly hampers and slows down its application, especially when we expect a quick assessment of the reliability of water supply with the required quality. Analyses of the needs in the reliability assessment of water supply of the required quality indicate that this methodology must be simplified.

In the work [47], the level of water quality certainty was assessed using probability theory. However, the authors did not focus on reliability assessment but on the development of a water quality assessment method. The said method consists of matching probability distributions with experimental data, and it refers only to microbiological parameters. To provide a model, the authors selected the negative binomial distribution, which they verified by means of the χ^2^ test. Yet, the authors advised being careful while interpreting the results of this probabilistic methodology for the assessment of water quality.

The considerations presented in [32] refer to the assessment of the quality of water treated at the treatment station before its introduction into the water supply network. The assessment method is based on the probability of exceeding the maximum values of water quality parameters. First, the reduction level of the concentration of a given pollutant is determined after a definite treatment process. Then, the models of damage events at various stages of water treatment are introduced, and in the final stage, the probability of exceeding the limit value for each quality parameter of the treated water is estimated.

To assess the reliability of water treatment, the authors of the work [45] introduced the notion of the indicator for surpassing the standard of a given water quality parameter as a criterion for reliability assessment. In addition, the surpassing levels of the normative values of water quality parameters, the maximum frequency of their occurrence, and the duration of these surpassing incidences were also arbitrarily adopted.

The considerations made so far have been mostly limited to the assessment of the quality of water produced at the treatment plant, and they do not fully resolve the problem involving the distribution reliability of treated water of acceptable quality. In [42], the said problem was addressed, yet the methodology of the reliability assessment process is quite extensive and requires the estimation of many specific reliability indicators.

In the present work, an attempt was made to build a mathematical model describing the reliability of water distribution in a water supply network in a city area, taking into account both the number and location of the measurement points of water quality parameters and the number and value of these parameters.

## 2. Methodology

The problem of assessing the degree of certainty of water supply of the required quality is complex, for example, due to the structure of the water supply system consisting of many elements (water intakes, water treatment stations, pumping stations, tanks, water supply) that can affect the quality of water supplied to recipients. It should be noted, however, that the water supply network has a special position in this structure. It is the last link in the serial structure of cooperation of these elements, providing water directly to consumers (water supply systems in buildings). In addition, the water supply network is characterized by a spatial arrangement and is generally very extensive. It functions in a specific area, which increases the potential threat to the quality of the distributed water. It is influenced by many factors related to the network itself, such as cable material, low water flow rates, stagnant water in the pipes, or neglect of network maintenance and operation. Considering the above, it was assumed that the water supply network is the most sensitive element of the water supply system due to water safety for consumers. Therefore, an emphasis was placed on developing a method for assessing the reliability of water supply with the required quality through a water supply network.

When developing the method, we allowed for the fact that water quality deterioration in the water supply system is mainly of a random nature. Hence, we took a probabilistic approach to assessing the degree of this certainty and used reliability theory for this purpose.

The method is based on data from water quality monitoring in the water supply network. The monitoring data area “batch” to the method. It should be noted that in relation to the assessment of the degree of certainty of water supply of the required quality, one can also talk about a model which is an algorithm assessing the degree of certainty of water supply with the required quality and thus has the hallmarks of assessing the reliability of the water supply.

In the following part, a simple method (model) of assessing the reliability of water supply with the required quality is presented, and the method is then demonstrated using the example of a real water supply network.

A degree of certainty to represent the reliability of water distribution of the required quality was defined for the purposes of modeling as the probability that the distribution system supplies water of the appropriate quality to the end user in a given period of its operation.

Since water quality is described by a specific set of parameters which must also be compliant with applicable standards [13,48], the probabilities of the distribution of water of the required quality should be estimated separately in terms of each parameter. The numerical values of individual water quality parameters are obtained by tests carried out at a specific set of research points (of the monitoring system) deployed in the area of the water supply network in operation. Therefore, in order to determine the global probability of the distribution of water of the required quality, the following sets are introduced:

—set of research points (observation points of the monitoring) (*P_m_*)
$i\in \left\{1,2,\ldots ,m\right\}=P_{m}$—*i*th research point
—set of water quality parameters (*W_n_*)
$j=\left\{1,2,\ldots ,n\right\}=W_{n}$—*j*th water quality parameter

where *m* is the number of research points, *n* is the number of investigated water quality parameters, and it is assumed that the evaluation concerns a fixed time interval.

The sets of research points and water quality parameters form a matrix field of research:

(1) $$P_{m}\times W_{n}∍(i,j)\mapsto X=\left[\begin{array}{l} X_{1}\\ X_{2}\\ .\\ .\\ .\\ X_{m} \end{array}\right]={\left[\begin{array}{cccccc} X_{11} & X_{12} & . & . & . & X_{1n}\\ X_{21} & X_{22} & . & . & . & X_{2n}\\ . & . & . & . & . & .\\ . & . & . & . & . & .\\ . & . & . & . & . & .\\ X_{m1} & X_{m2} & . & . & . & X_{mn} \end{array}\right]}_{m\times n}\overset{zapis}{=}{\left[X_{ij}\right]}_{m\times n},$$

where *X_ij_* is a random (one-dimensional) variable defining the value of the *j*th water quality parameter at the *i*th research point of the known distribution function $F_{ij}(x):=P(X_{ij}\leq x),x\in R$ and *X_i_ = (X_i_*_1_, *X_i_*_2_, …, *X_in_*) is an *n*–dimensional random variable defining the values of water quality parameters at the *i*th research point of the distribution function given by the formula

(2) $$F_{i}(x_{1},x_{2},\ldots ,x_{n}):=P(X_{i1}\leq x_{1}\wedge X_{i2}\leq x_{2}\wedge \ldots \wedge X_{in}\leq x_{n}).$$

Assuming the stochastic independence of random variables *X_i_*_1_, *X_i_*_2_, …, *X_in_*, we obtain

(3) $$F_{i}(x_{1},x_{2},\ldots ,x_{n})=F_{i1}(x_{1})\cdot F_{i2}(x_{2})\cdot \ldots \cdot F_{in}(x_{n})=\prod_{j=1}^{n}F_{ij}(x_{j}).$$

If we let *s_j_* denote a standard (critical, unsurpassable, normative) value of the *j*th water quality parameter, then the probability of normative water quality at the *i*th research point is

(4) $$P_{i}=F_{i}(s_{1},s_{2},\ldots ,s_{n})=\prod_{j=1}^{n}F_{ij}(s_{j}),$$

and assuming the independence of the tests on water quality parameters, the probability of normative water quality in the entire distribution system, i.e., the reliability of water distribution of the required quality, assumes the following form:

(5) $$P=\prod_{i=1}^{m}P_{i}=\prod_{i=1}^{m}\prod_{j=1}^{n}F_{ij}(s_{j}).$$

The estimator of the probability of the normative value of the *j*th water quality parameter at the *i*th research point, determined on the basis of the monitoring data, is expressed by

(6) $$F^{*}{}_{ij}(s_{ij})=1-\frac{k_{ij}}{K_{ij}}$$

where F*_ij_ is the empirical probability of non-surpassing the required level of the *j*th water quality parameter at the *i*th research point within a given time interval; *k_ij_* is the number of results of water quality tests obtained at the *i*th research point out of *K_ij_* which do not comply with the required water quality on the basis of the *j*th quality parameter *x*; and *K_ij_* is the number of all test results of water quality on the basis of the *j*th quality parameter taken at the *i*th research point.

The proposed model can be applicable for the assessment of reliability taking into account different sets of water quality parameters. Thus, it can assess the reliability of water distribution with the complete required quality, i.e., the probability ensuring all water quality parameters, or the reliability of the distribution of water of incomplete required quality, e.g., the probability of physicochemical water quality assurance or the probability of microbiological water quality assurance.

The model can also describe the reliability of ensuring the required water quality in the whole area of its operation, in a part of this area (e.g., a separate water supply zone, housing estate, commercial and service center, etc.), or at a given point of the network (at the water supply place, e.g., hospital, leisure center, etc.).

## 3. Results and Discussion

**Figure 1:**
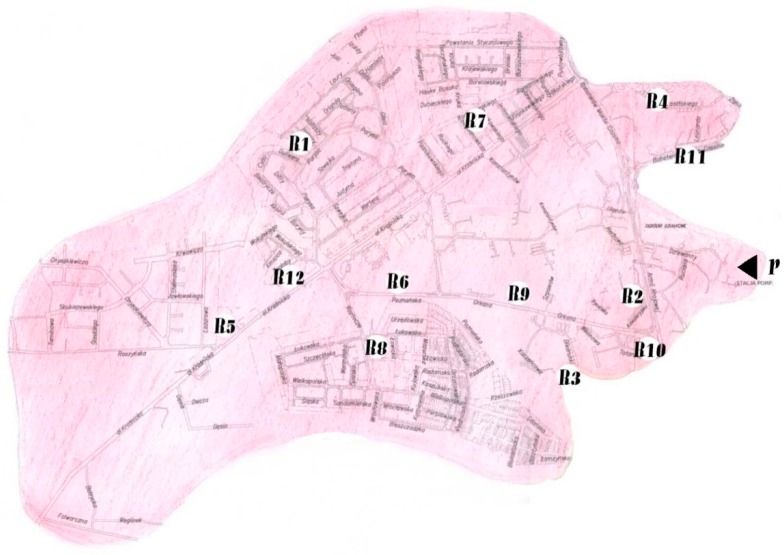
Distribution of water quality monitoring points in the analyzed area of the distribution system. R1, R2, …, R12—points of water sampling for testing, ◄ P—supplying the zone with water (pumping station).

The application method of the model is illustrated in the example of the probability assessment of the distribution of water of the required microbiological quality, which determines the certainty of water supply to consumers. The data for the reliability assessment were obtained from a water quality monitoring system in areal water supply network which is one separate zone of a large water supply system. The zone is fed from its own intake of underground water collected by means of drilled wells and has an independent water treatment station and a pumping station. The water supply network in the analyzed zone supplies water to consumers in the amount of approximately 25,000 m^3^/day. In this area, water quality indicators are monitored at 12 points. The distribution of the water quality testing points is shown on the city map in Figure 1.

The results of microbiological tests from a period of 5 years were used. The example was used to estimate the probability of the distribution of water with the required microbiological quality in relation to the standards included in [48] (Table 1), which also correlate with the requirements of the Directive [13].

In order to determine the probability of the distribution of water of the required microbiological quality, the empirical probabilities of the normative values of the water quality parameters listed in Table 1 at each of the 12 points according to Equation (6) were estimated first. The results are presented in Table 2.

Using the calculation results from Table 2, the probability of water distribution of the required microbiological quality was calculated, adopting for this purpose Equations (4) and (5). As a result, a probability value of 0.956 was obtained, which means that the probability of not ensuring the required microbiological quality within 5 years is 0.035. This value could also be interpreted as a margin of operational risk of not meeting the microbiological water quality required by the WHO Guidelines [48].

The analysis shows that the reliability of water distribution of the required microbiological quality was determined by the number of fecal coliform bacteria. The value of this parameter was not compliant with the required standard in one test.

The occurrence of this unfavorable phenomenon can be attributed principally to an insufficient concentration of disinfectant (chlorine) in the water, which could have safeguarded it against secondary contamination. The recorded concentrations of this disinfectant at the tested points were often lower than the normative value (residual concentration of chlorine 0.3 mg/L) [48].

The obtained reliability of water distribution of the required microbiological quality is associated with the maintenance of a specific standard—in this case, the WHO standard. A similar assessment of water supply certainty can be made with respect to other standards using the proposed method.

## 4. Conclusions

1. The analysis of the results of water quality tests in water supply networks demonstrates that the changing values of parameters describing this quality often do not comply with the applicable standards, posing a serious threat to the health safety of water. To assess the effects of this phenomenon, a method of assessing the reliability of water distribution with the required quality was proposed. This method uses the results of water quality tests obtained from the monitoring of the water supply network. Such monitoring is carried out in the majority of water distribution networks.

2. The method is dedicated to assessing the degree of ensuring the required water quality or reliability of water supply through the water supply network, taking into account different sets of water quality indicators. It allows us to assess the reliability of water supply with the required quality, e.g., microbiological, physical, or chemical. Depending on the needs, it is also possible to assess the reliability of ensuring the required water quality in the whole area of the settlement unit or in a separate part of the area, e.g., a housing estate, or in a selected facility, e.g., a hospital.

3. The results obtained in the calculation example in which data from areal water supply network were used demonstrate that there is a certain risk margin of not ensuring the quality of water required by the applicable standards. The open question is, therefore, to establish an acceptable level of that risk.

## Figures and Tables

**Table 1:** (Normative) values of microbiological water quality parameters according to WHO Guidelines [48] used in the calculation example.

Water Quality Parameter	Value of Water Quality Parameter
Number of coliform bacteria in 100 mL of water not higher than (LBC)	0
Number of fecal coliform bacteria in 100 mL of water not higher than (LBCK)	0

**Table 2:** Estimation of empirical probability estimators *F_ij_* of the normative value of the *j*th microbiological parameter of water quality at the *i*th research point according to WHO standards.

Research Point “I”	Number of Coliform Bacteria in 100 mL of Water (*j* = 1)			Number of Fecal Coliform Bacteria in 100 mL of Water (*j* = 2)		
	A	*N*	*F_ij_*	A	*N*	*F_ij_*
1	25	0	1	25	0	1
2	27	0	1	27	0	1
3	21	0	1	21	0	1
4	44	0	1	44	0	1
5	25	0	1	25	0	1
6	2	0	1	2	0	1
7	23	0	1	23	1	0.956
8	25	0	1	25	0	1
9	1	0	1	1	0	1
10	28	0	1	28	0	1
11	1	0	1	1	0	1
12	2	0	1	2	0	1

A—number of all test results within the time period of 5 years; *N*—number of test results noncompliant with standards; *F_ij_*—probability estimated from Equation (6).

## Research Impact Statement

The presented model is a significant contribution to the development of advanced methods to be used to assess the reliability of water supply taking into account different sets of water quality parameters.
